# Space Charge Behavior of Thermally Aged Polyethylene Insulation of Track Cables

**DOI:** 10.3390/polym14112162

**Published:** 2022-05-26

**Authors:** Zhichao Qiao, Wangsong Wu, Zhaowei Wang, Ling Zhang, Yuanxiang Zhou

**Affiliations:** 1 CRSC Research and Design Institute Group Co., Ltd., Beijing 100070, China; qzc_11@163.com; 2 State Key Laboratory of Power System, Department of Electrical Engineering, Tsinghua University, Beijing 100084, China; wuws19@mails.tsinghua.edu.cn; 3 Wuwei Electric Power Supply Company of the State Grid, Wuwei 733000, China; zw_wang2019@163.com

**Keywords:** multi-layer insulation, polyethylene, space charge, thermal aging, tensile strength, oxidation induced temperature

## Abstract

The interface of multi-layer insulation is a relatively weak point in the cable system during the long-term high-temperature service. Space charge is prone to continuously accumulate in the interface area, leading to the deterioration of electrical properties and even insulation failure in advance. The knowledge about thermal oxidation of polyethylene (PE) materials at the molecular level is still urgent to explore. Herein, single-layer and double-layer PE insulation, representing the typical insulation structure of frequency-shift pulse voltage track cables, were prepared and thermally aged in the oven for up to 360 h. Thermal, mechanical, electrical, and space charge characterizations were systematically carried out. Thermogravimetric analyzer and oxidation induced temperature (OIT) measurements confirmed that LDPE’s thermal-oxidative aging temperature range was the lowest among the three PE groups in the O_2_ atmosphere. After 360 h thermal aging, the tensile property of HDPE material kept relatively stable, while the elongation at break of the other two groups was lower than 50%. Unaged HDPE exhibits apparent charge injection and migration, leading to the severe electric field distortion of 20%. Noticeable charge accumulation can be observed at the unaged double-layer sample interface due to the mismatching of DC conductivity, which play a significant role in the aged double-layer samples. This work utilizes precise thermal analysis to provide new information about the resistance ability of thermal oxidation of LDPE, FPE, and HDPE, and its influence on space charge behaviors, which is helpful for the insulation design and evaluation in cable applications.

## 1. Introduction

Polyethylene (PE) materials are widely used in cable insulation, which involves the application of multi-layer insulation structure. [1]. For example, the insulation layer of frequency shift pulse track cable involves “skin-foam-skin” three-layer insulation structure. [2,3]. Owing to many interfaces exist in multi-layer insulation, the process of charge transport becomes more complicated [4,5,6], especially under the operating conditions of high temperature or temperature gradient, the insulation material will undergo thermal aging, and the interface is more likely to cause insulation defects, resulting in deterioration and failure of electrical and mechanical properties [7,8]. Therefore, it is necessary to comprehensively study the differences in thermal mechanical, and electrical properties between single-layer and multi-layer insulation structures considering thermal aging and summarize the critical indicators for the aging evaluation [9].

The space charge development is known to have a critical impact on the lifetime and the evaluation of the aging degree of insulation materials under high DC stress [10]. Its transport is closely associated with hopping charge carriers [11,12,13]. While the thermal aging process will significantly change the trap characteristics of polymer materials, which directly leads to different space charge behaviors [14,15]. Therefore, space charge distribution could be used to reveal the trap property and evaluate the aging degree [16].

Loads of work have been carried out to characterize single-layer insulation’s space charge behavior and analyze the internal trap characteristics under thermal aging condition [17]. Dakka et al. [18] measured the space charge characteristics of crosslinked polyethylene (XLPE) materials and found that in the first few hours of thermal aging, the accumulation rate of space charge was related to the breakdown time of the insulation layer. The higher the initial charge accumulation, the shorter the insulation failure time. Hozumi et al. [19] also measured the polarity induced space charge of XLPE AC cable samples with different aging degrees. They found that the induced space charge increased with the aging duration, and the induced charge lasted longer after polarization. In contrast, the total space charge was related to the AC breakdown strength and other parameters. However, the effect of thermal aging on space charge characteristics of multi-layer insulation structures is not sufficiently studied [20,21,22].

Moreover, the changes in internal molecular structure caused by thermal aging are irreversible for insulation materials. Meanwhile, the physicochemical, and electrical properties will also vary with the change in the molecular structure of the polymer materials [23,24,25,26]. These properties and space charge characteristics are seldom considered comprehensively [27,28,29], which makes it challenging to evaluate the aging degree accurately.

This work studies the effect of thermal oxidation aging on the typical single-layer and double-layer insulation of frequency shift pulse track cables, including thermal, mechanical, electrical, and space charge behaviors. Two precise thermal analysis methods were utilized for extracting and evaluating the difference in resistance ability of thermal oxidation among PE materials. Based on the thermal properties, the influence of the thermal degree of PE materials on the other properties is summarized.

## 2. Materials and Methods

**Figure 1:**
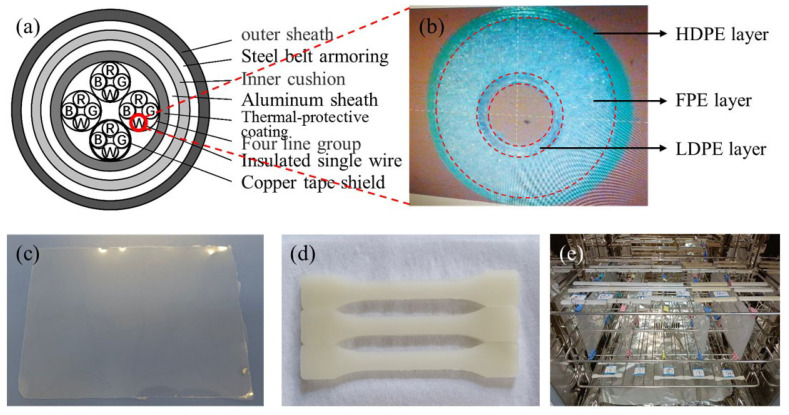
Structure and sample diagram (**a**) the structural of frequency-shift pulse voltage track cable, (**b**) the microscope diagram of “skin-bubble-skin” insulation layer, (**c**) PE film sample, (**d**) dumbbell-shaped samples, and (**e**) sample placement in the thermo-oxidative aging oven.

Low-density polyethylene (LDPE) pellets have a density of 0.925 g/cm^3^ and a melt index 0.4 g/10 min. Foamed polyethylene (FPE) pellets have a density of 0.948 g/cm^3^ and a melt index of 3.9 g/10 min. High-density polyethylene (HDPE) pellets have a density of 0.945 g/cm^3^ and a melt index of 0.8 g/10 min. All LDPE, FPE, and HDPE materials were extruded into multi-layer insulation, called the “skin-bubble-skin” structure, in the frequency-shift pulse voltage track cables (see Figure 1a,b), and provided in the pellet form by Henan Jiaozuo Railway Electric Cable Company (Henan, China). As a reference, 100 μm-thick LDPE films without additives were purchased from Goodfellow Company (London, UK), denoted as Ref-LDPE.

This paper uses a SCM20 film blowing machine and a SZS-20 injection molding machine (Wuhan Ruiming Experimental Instrument Manufacturing Co., Ltd., Wuhan, China) to mass produce PE films with uniform thickness dumbbell-shaped samples (see Figure 1c,d). Films with different thicknesses (e.g., ~120 μm, ~80 μm, and ~40 μm) can be obtained by controlling the film blowing machine’s motor speed and outlet temperature. The size of the dumbbell-shaped sample for the tensile test is 75 mm × 10 mm × 2 mm. Considering the heat resistance of PE materials, the thermal aging temperature of Ref-LDPE and LDPE samples was set to 90 °C, while that of FPE and HDPE samples was set to 100 °C (see Figure 1e). At 10 h, 100 h, and 360 h, a certain number of film samples were taken out from the oven for tests.

A TA Q250 Differential Scanning Calorimetry (DSC) was used to evaluate the energy absorbed or released by PE samples to evaluate melting and crystallization performance in an N_2_ flow or oxidation performance in an O_2_ flow. First, ~10 mg pellets were put into a DSC aluminum pan for melting and crystallization measurements. The test temperature range was set as 0–180 °C, and the heating and cooling rates were set as 20 °C/min. To clear thermal history, heat and cool the sample twice, and only the second-round test result was adopted. Based on the ISO 11357-6:2008 Standard, the oxidation induced temperature (OIT) test based on DSC was carried out. ~10 mg pellets were linearly heated to 320 °C in the O_2_ atmosphere with a ramp rate of 20 °C/min, and the heat flow data of the oxidation reaction was recorded.

The thermal decomposition characteristics of PE samples were investigated by a TA Q55 thermogravimetric analyzer (TGA) (New Castle, DE, USA) both in N_2_ and O_2_ atmospheres. TGA has really high precision of mass up to 10 ng. For the N_2_ atmosphere, ~10 mg pellet sample were prepared and placed in the platinum pan. The temperature was controlled at 30–800 °C with a ramp rate of 20 °C/min to record the mass loss due to the bond break and volatilization in PE chains. For the O_2_ atmosphere, the resistance ability of thermal oxidation of PE materials can be judged by observing the thermal oxidation and decomposition reaction during the linear heating process accompanied by the mass change. The O_2_ flow rate was set as 60 mL/min, the heating rate 20 °C/min, and the test temperature range 30–600 °C.

According to the ASTM D638 Standard, the tensile test was carried out using an MIT-50 electronic universal testing machine (Changzhou Sanfeng Instrument Technology Co., Ltd., Changzhou, China). The dumbbell-shaped specimen was selected with the middle width of 5 mm. The initial distance between fixtures was 50 mm, and the tensile speed was 50 mm/min.

According to the IEC 60093:1980 Standard, the DC conduction current of PE film samples was measured at room temperature with a laboratory-made three-electrode apparatus and a high-resolution Keithley 6517B electrometer. The film thickness was ~120 μm, and the size was 50 mm × 50 mm. The polarization time under 10 kV/mm was set to 1200 s, which was regarded long enough to achieve a quasi-steady state.

The AC breakdown strength (BDS) test adopted sphere-plate electrodes and was carried out in the transformer oil with a ramp rate of 0.5 kV/s at room temperature. The thickness of film samples was ~120 μm. Before the test, all samples were wiped with alcohol to remove surface impurities and then dried in a vacuum oven for 24 h at 60 °C to eliminate moisture interference.

The space charge characteristics of single-layer and double-layer PE insulation were studied using the pulsed electroacoustic (PEA) method at room temperature. The applied electric field was set as −50 kV/mm. Specifically, the critical parameters of a high-voltage pulse generator include the output voltage of 900 V, the pulse width of 4.5 ns, the frequency of 1000 Hz, and the average waveform acquisition interval of 5 s. In this work, the anode represented the ground electrode, and the cathode represented the electrode with a negative DC voltage.

## 3. Results and Discussion

### 3.1. Thermal Property

**Figure 2:**
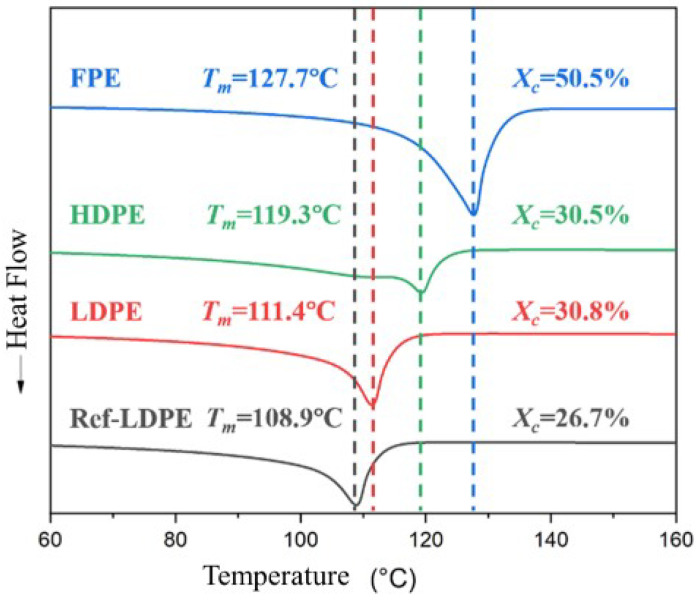
DSC curves of unaged PE samples in the N_2_ atmosphere.

The DSC curves of unaged Ref-LDPE, LDPE, FPE, and HDPE samples in the N_2_ atmosphere are shown in Figure 2. The melting point of ref-LDPE and LDPE is low, while FPE has the highest melting point of 127.7 °C and crystallinity of 50.5%.

**Figure 3:**
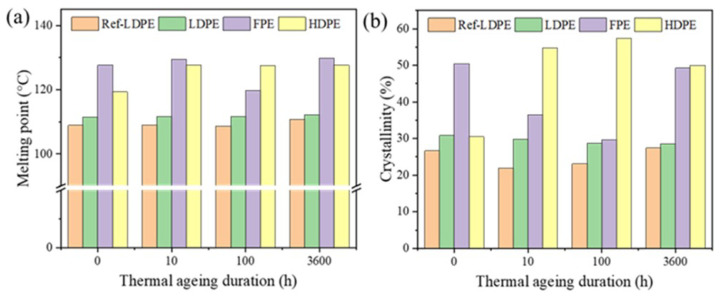
(**a**) Melting point and (**b**) crystallinity of PE samples with different thermal aging durations.

After thermal aging treatment for 10 h, 100 h, and 360 h, various samples’ melting point and crystallinity changed, as shown in Figure 3. The melting point of REF-LDPE and LDPE is lower than FPE and HDPE (see Figure 3a). With the extension of thermal aging duration, the melting point of FPE and HDPE changes more obviously than that of RE-LDPE and LDPE. Notably, the melting points of HDPE after a thermal aging increase by ~7% more than that of the unaged samples.

Similarly, the crystallinity of LDPE and Ref-LDPE keep stable with the thermal aging duration (see Figure 3b). In contrast, the crystallinity of FPE and HDPE exhibits a noticeable change with the thermal aging duration. Specifically, an evident increasing trend was observed for HDPE, which may be due to a recrystallization process during the thermal aging treatment. The crystallinity of FPE drastically decreased after thermal aging, which may be due to the decomposition of materials under thermal aging. However, when the aging duration increased to 360 h, the crystallinity of FPE recovered to the level before thermal aging, which may be due to the formation of new phases.

**Figure 4:**
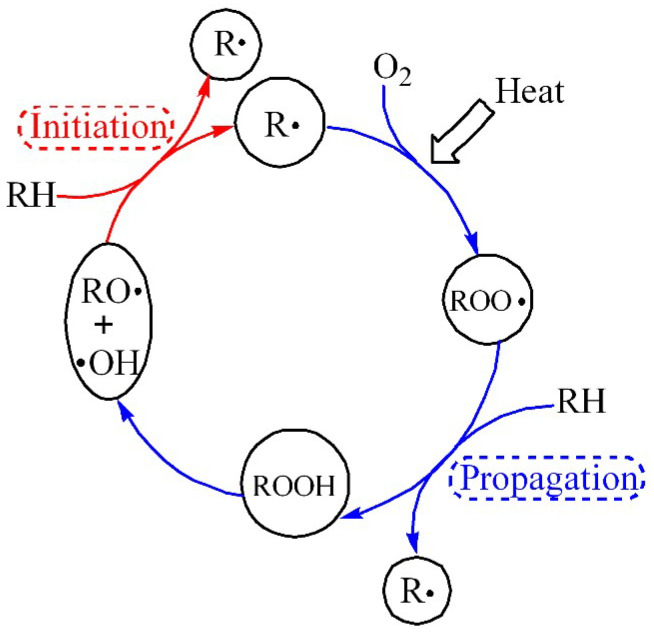
Initiation and propagation of thermal oxidation of PE chains under heat and O_2_.

During regular service, the cable will inevitably contact the air, and the polymer insulation material will combine with O_2_ molecules to form oxygen-containing groups, which will lead to thermal oxidation degradation. The polymer’s electrical, mechanical, and thermal properties will decline to different degrees, affecting the regular use of the cables. Figure 4 shows the initiation and propagation of thermal oxidation (RH represents PE chains) under the high temperature and O_2_ atmosphere. The oxidative degradation process of PE materials is a chain reaction process, which includes chain initiation of primary free radicals, chain growth of oxidized products, and chain termination of free radicals.

**Figure 5:**
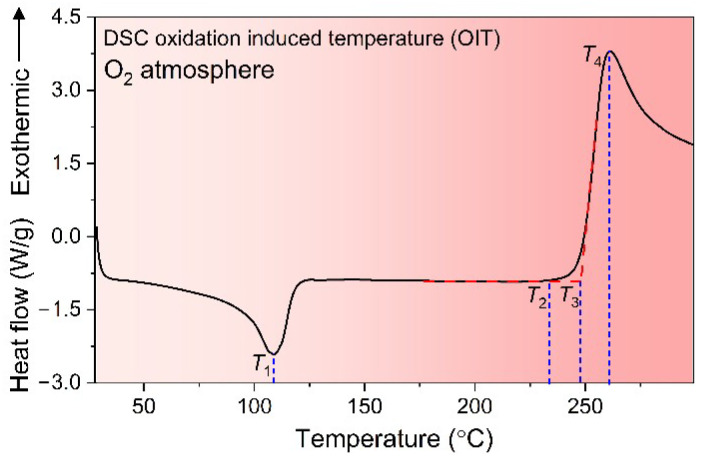
Schematic diagram of OIT curve of XLPE material based on the DSC technique.

Figure 5 takes an OIT curve of XLPE material, for example. Under O_2_ conditions, the DSC aluminum pan rises from 30 °C to 320 °C at a rate of 20 °C/min, with a melting temperature of *T*_1_ around 110 °C. Since the oxidation process is an exothermic reaction, the heat flow at 230 °C gradually increases with *T*_2_ as the initiation temperature for oxidation, *T*_3_ as the oxidation induced temperature (OIT) (the intersection of tangent laws), and *T*_4_ as the peak temperature for oxidation.

**Figure 6:**
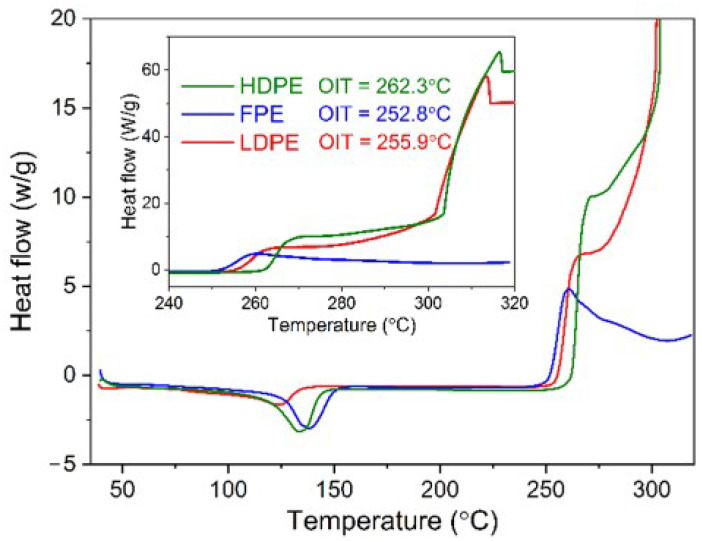
DSC OIT curves of LDPE, FPE, and HDPE materials.

Figure 6 shows the OIT curves of three PE materials, illustrating the heat flow at high temperatures. According to the above, there is a difference in OIT between three PE materials, i.e., HDPE is the highest and FPE is the smallest, with a gap of ~10 °C. Notably, the FPE material oxidizes at 252.8 °C and reached its first oxidation peak at ~260 °C. However, the subsequent heat flow of the oxidation reaction showed a continuous downward trend, with no second prominent exothermic peak before 320 °C, as seen in HDPE and LDPE groups (see the illustration in Figure 6). This means that a second oxidative exothermic peak in FPE should occur at temperatures above 320 °C, pending further validation by subsequent TGA measurements. Figure 6 also shows the absorption peaks of the melting process of three PE materials at 100–150 °C, consistent with DSC test results in the N_2_ atmosphere.

**Figure 7:**
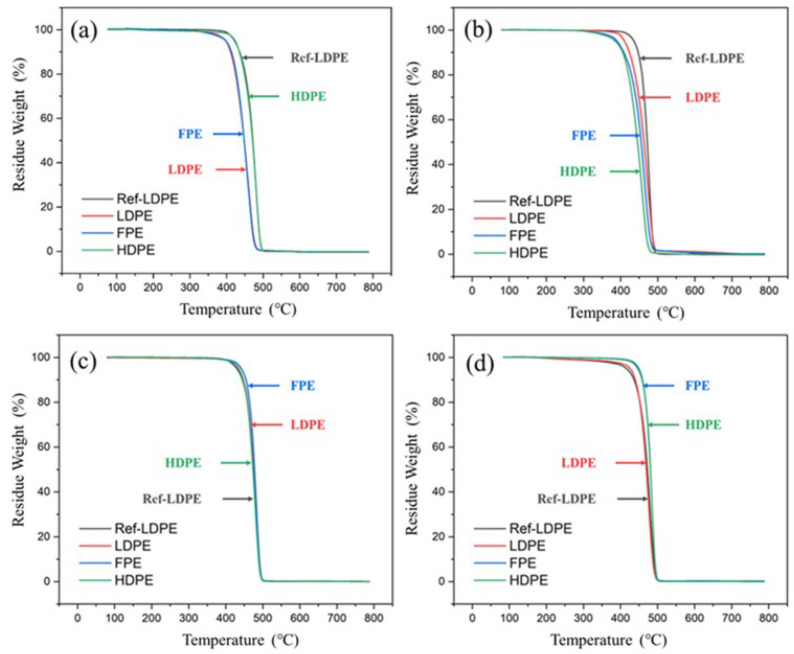
TGA curves of unaged PE samples in the N_2_ atmosphere with different thermal aging durations. (**a**) 0 h, (**b**) 10 h, (**c**) 100 h, and (**d**) 360 h.

Figure 7 shows that the TGA curves of all PE materials are relatively similar, corresponding to the fastest decomposition (fastest mass decline), indicating the similar thermal stability of molecular chains. In addition, there is only one stage of weight loss in the TGA curve of each PE material, meaning that there is only one principal component.

**Figure 8:**
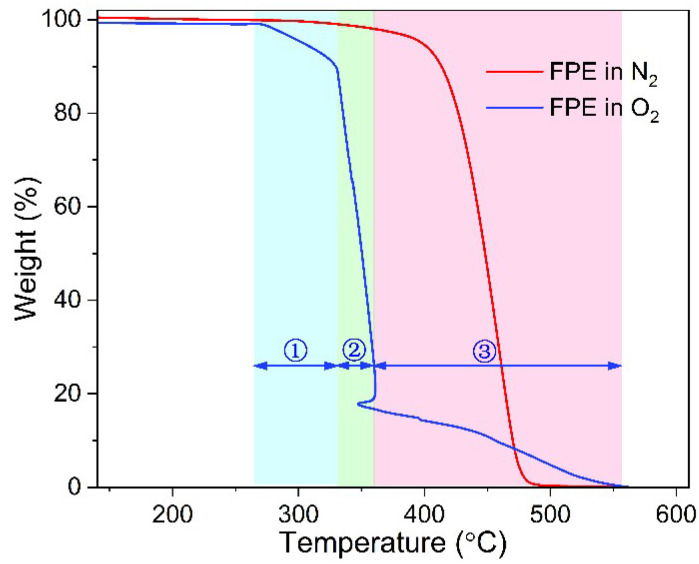
Comparison of TGA curves of FPE material in N_2_ and O_2_ atmospheres.

Figure 8 compares the effects of N_2_ and O_2_ on the decomposition curves of FPE material via TGA measurements. The weight loss curve of FPE can be divided into three stages under the O_2_ atmosphere. The first stage is 270–330 °C with a weight loss of 10% due to the thermal-oxidation aging, and the second stage is 330–360 °C with a weight loss of 73% due to C−C molecular chain pyrolysis. The third stage is 360–560 °C, with a weight loss of 17% due to the C−O groups. In contrast, FPE material has only one weight-loss stage in the N_2_ atmosphere with a temperature range of 400–480 °C. The thermal weightlessness in the N_2_ atmosphere is only C−C molecular chain pyrolysis. Therefore, the thermal weight loss in the first stage of FPE under an O_2_ atmosphere is closely related to the thermal aging process during actual service.

**Figure 9:**
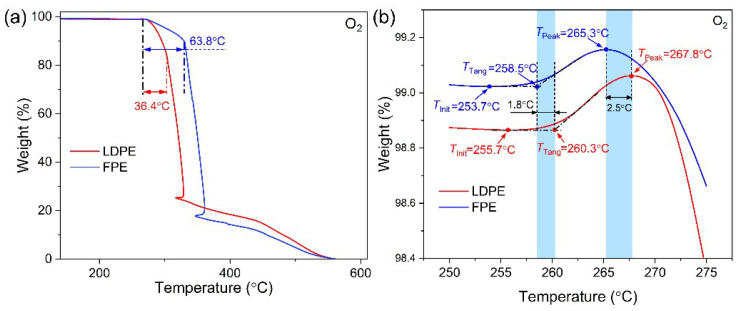
Thermal degradation curves of FPE and LDPE in TGA tests under O_2_ atmosphere (**a**) 40–600 °C, (**b**) 250–275 °C.

FPE molecules undergo a slower chain break during thermal oxidation in the first stage than LDPE as shown in Figure 9a. This phenomenon might be due to a foaming structure in FPE that causes the production of fewer free radicals in the presence of O_2_, which slows down the automatically accelerated oxidation process, increasing the temperature at which subsequent oxides begin to break down by 27.4 °C. Figure 9b is a partial magnification of Figure 9a. The initial temperature of thermal oxidation aging in LDPE is 2 °C higher than that in FPE, the nodal point of tangent during the ascent stage is 1.8 °C higher, and the peak temperature of oxidation is 2.5 °C higher. The previous DSC OIT curve of LDPE was 3.1 °C higher than FPE. Moreover, it is worth mentioning that in Figure 6, LDPE and HDPE materials have a second significant peak of oxidative exothermic activity between 310–320 °C, whereas FPE material have not yet occurred. It can be inferred from Figure 9a above that a second significant exothermic peak of FPE material may occur between 330–340 °C.

Table 1 compares DSC OIT and TGA parameters for PE materials in an O_2_ atmosphere. TGA observes the mass change with temperature during thermal aging, decomposition, and volatilization. DSC reflects the energy change of the samples with a linear temperature increase. In particular, both methods reflect the thermal aging process of PE materials in the temperature range of 200–320 °C. However, due to the different sensing mechanisms, system sensitivity, and measurement resolution, the difference of corresponding temperature on the curves of the two methods needs to be combined with the physical process of thermal oxidation reaction to give a reasonable interpretation. Take the *T*_3_ temperature on the DSC OIT curve, representing the temperature at which the oxidation reaction begins. The rapid increase in the heat flow towards the exothermic direction also reflects a slight increase in the sample mass on the TGA curve (see *T*_Init_ in Figure 9b). Therefore, *T*_3_ in DSC OIT can be matched to *T*_Init_ in TGA curves for FPE material with a bias of less than 1 °C (i.e., 252.8 °C vs. 253.7 °C). From the TGA curve, Ref-LDPE has a much lower thermal oxidation temperature (~50 °C) compared to the others, which might mean that all Jiaozuo LDPE, FPE, and HDPE materials should have some content of antioxidant.

### 3.2. Mechanical Property

**Figure 10:**
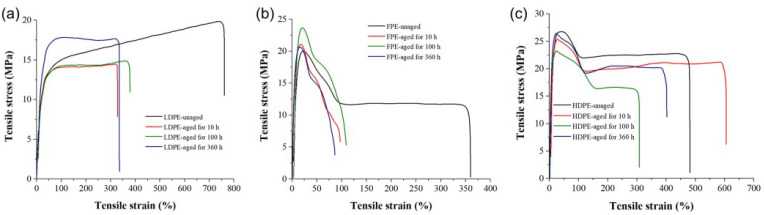
Tensile curves of thermally aged PE samples. (**a**) LDPE, (**b**) FPE, and (**c**) HDPE.

**Figure 11:**
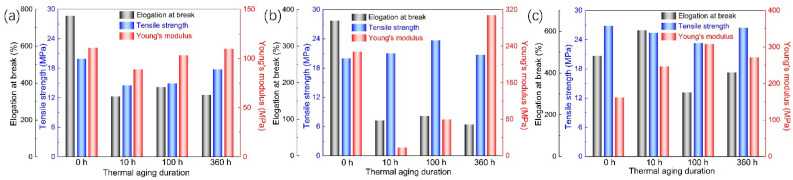
Tensile parameters of thermally aged PE samples. (**a**) LDPE, (**b**) FPE, and (**c**) HDPE.

The tensile stress-strain curves of three thermally aged PE materials are shown in Figure 10. According to the curves, the tensile parameters (elongation at break, tensile strength, and elastic modulus) are deduced, and the results are shown in Figure 11. It can be seen that the elongation at the break of LDPE and FPE in the innermost layer of the “skin-foam-skin” structure decreases after thermal aging. In contrast, the elongation at the break of HDPE in the outermost layer experiences first increases and then decreases with the aging duration. Therefore, thermal aging reduces the tensile ductility of three PE materials. The results of elastic modulus after thermo-oxidative aging for 360 h also show that PE materials become brittle under long-term high temperatures.

The tensile properties of the three PE materials deteriorate with the aging duration, which could be attributed to microscopic physical and chemical defects. Therefore, the service life of the insulation materials is reduced compared with the initial status, and the probability of mechanical or electrical failure is gradually increased. The elongation at the break of FPE decreases to ~100%, indicating that its life is shortened more sharply than those of LDPE and HDPE. In the practical application, because the FPE layer is in the middle of the “skin-foam-skin” triple-layer insulation, it is relatively less affected by the temperature gradient of the external environment. Still, many loose microporous structures generated by internal foaming are conducive to the oxidation reaction. Thermal aging is easy to occur, leading to the deterioration of insulation performance.

### 3.3. Electrical Property

**Figure 12:**
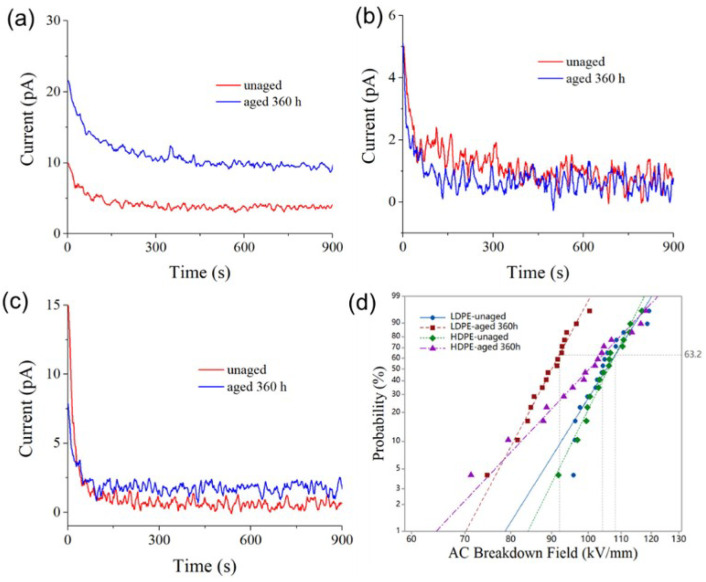
Conduction current of (**a**) LDPE, (**b**) FPE, and (**c**) HDPE under 10 kV/mm. (**d**) Weibull plots of AC BDS.

Figure 12 shows the electrical conduction current and AC BDS results of thermally aged PE materials. Conduction current measurements were performed with a 10 kV/mm electric field. The steady-state current was smaller than 5 pA for unaged LDPE material, but larger than 10 pA after thermal aging, as shown in Figure 12a. As can be seen from Figure 12b,c, the conduction currents of FPE and HDPE materials do not change significantly after thermal aging, and the steady-state currents are both lower than 2 pA. Among the three PE materials, aged LDPE material has the most considerable conduction current, suggesting that thermal oxidation aging has the most significant effect on the internal microstructure of LDPE material, introducing more shallow traps, providing more channels for carrier transport, and thus increasing electric conductivity. Further, the AC BDS of aged LDPE material decreased significantly by 15% in Figure 12d, while that of HDPE material only decreased by 4%.

### 3.4. Space Charge Behavior of Single-Layer Samples

**Figure 13:**
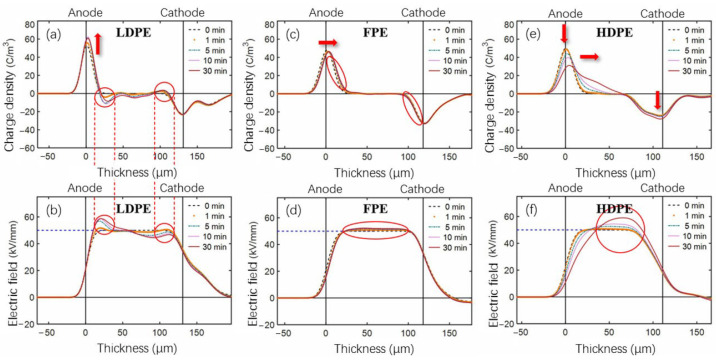
(**a**,**c**,**e**) Space charge and (**b**,**d**,**f**) electric field distribution of single-layer unaged LDPE, FPE, and HDPE samples polarized for 30 min under −50 kV/mm, respectively.

A −50 kV/mm electric field was applied to unaged single-layer PE film samples, and the internal space charge and electric field distribution were obtained [30] as shown in Figure 13. Two red circles in Figure 13a represent the accumulation of space charges in LDPE samples near the anode and cathode, respectively. As the polarization time increases, heterocharge accumulation occurs in the vicinity of both electrodes. The charge amount near the anode is more significant than that at the cathode, so positive electrode charges are induced at the anode, as indicated by the arrow in Figure 13a. This result is also reflected in the electric field distribution in Figure 13b, which shows an increase of the electric field near the anode. Although the heterocharge accumulation also exists near the cathode, the aberration effect of the electric field decreases due to the offset of the negative charge near the anode.

FPE material showed a small charge injection and migration to the bulk of the sample in both cathode and anode regions, as shown by the arrows and circles in Figure 13c. A slight distortion of the electric field in the bulk area is shown in Figure 13d. In Figure 13e, the space charge distribution of HDPE material shows that the negative charge density peak has a different shape than the right peak at the initial polarization stage. The charge in the anode region is injected and migrated inwards with the polarization, and some charge accumulates near the cathode. As can be seen from Figure 13f, the space charge behavior above also leads to a significant electric field distortion.

**Figure 14:**
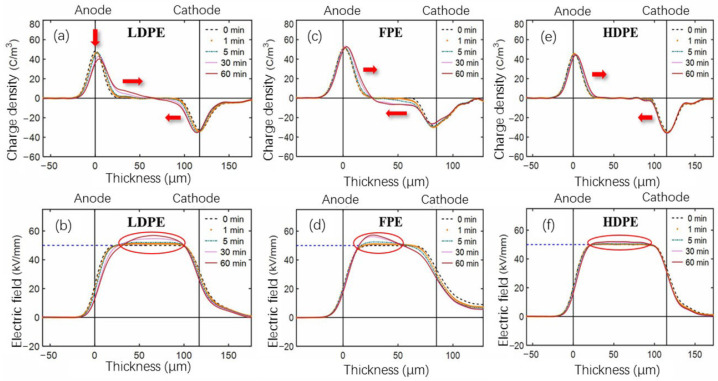
(**a**,**c**,**e**) Space charge and (**b**,**d**,**f**) electric field distribution of single-layer 360 h aged LDPE, FPE, and HDPE samples polarized for 60 min under −50 kV/mm, respectively.

Figure 14 shows the space charge and electric field distribution of three single-layer PE materials after 360 h aging. During polarization, a significant accumulation of homocharge occurred near the electrode in LDPE film samples, and the accumulated charges migrated further into the bulk than in unaged samples, as shown in Figure 14a. Figure 14b shows that there is also a significant electric field distortion. Compared to the unaged group, the negative charge of FPE material is injected from the cathode in large quantities and migrates to the anode. Meanwhile, the positive charge near the anode also migrates slightly inward, as shown in Figure 14c. Figure 14d depicts heterocharge near the anode sensing a large electric field distortion than unaged FPE films. Figure 14e,f shows that space charge evolution and electric field distribution aberrations in aged HDPE material are insignificant.

**Figure 15:**
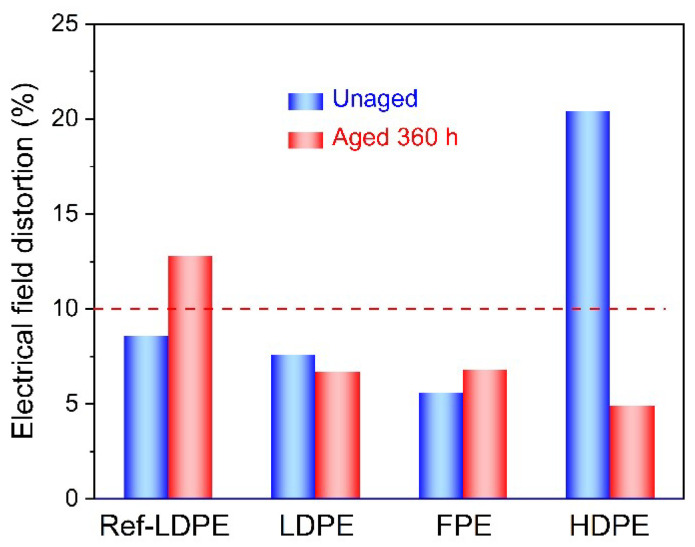
Electric field distortion of single-layer samples polarized for 30 min under −50 kV/mm.

Figure 15 shows that the unaged HDPE has the largest electric field distortion of the three PE materials, while the aged HDPE has the smallest. Therefore, more deep traps may be introduced into HDPE material after thermal oxidation aging, contributing to the inhibition of space charge. Combined with the above thermal and fundamental electrical performance analysis, HDPE has better resistance ability of thermal oxidation aging than LDPE and FPE.

### 3.5. Space Charge Behavior of Double-Layer Samples

A double-layer structure is formed by the physical pressure of films (i.e., two films are dripped with silicone oil). In the PEA measurement, according to the different materials in contact with anode and cathode (P represents anode and N represents cathode), the double-layer structure can be denoted as LDPE(P)/FPE(N) and HDPE(P)/FPE(N).

**Figure 16:**
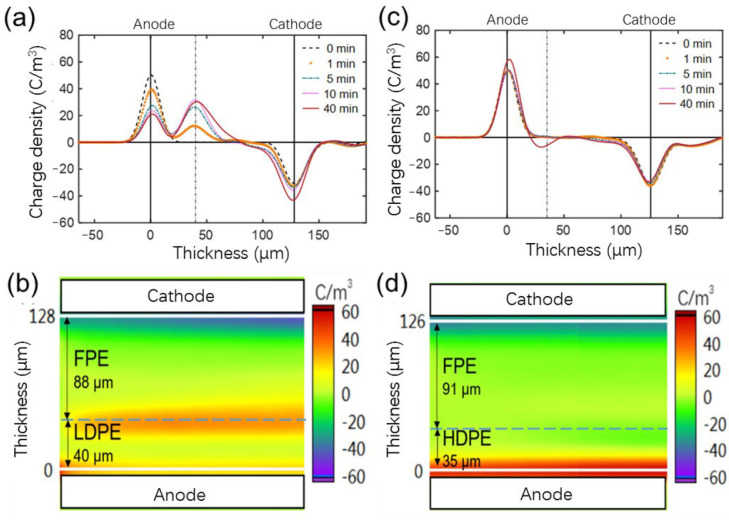
Space charge distribution of unaged (**a**,**b**) LDPE(P)/FPE(N) and (**c**,**d**) HDPE(P)/FPE(N) polarized for 40 min under −50 kV/mm.

The space charge and electric field distribution of unaged LDPE(P)/FPE(N) and HDPE(P)/FPE(N) double-layer samples during 40 min polarization under −50 kV/mm are shown in Figure 16. A large amount of positive charge injection occurred in the area near the anode of LDPE(P)/FPE(N) double-layer sample during the 40 min polarization in Figure 16a, while a large amount of negative charge accumulated near the cathode, and a large amount of positive charge accumulated in the middle interface area. In the two-dimensional color diagram of Figure 16b, the charge density of dark color appears at the interface between the FPE layer and the LDPE layer. It can be seen from Figure 16c that during the 40 min polarization, a small number of negative charges were injected in the area near the anode of the HDPE(P)/FPE(N) sample, and some negative charges were injected near the cathode. On the other hand, the interface is characterized by light charge density in the 2D color diagram of Figure 16d.

**Figure 17:**
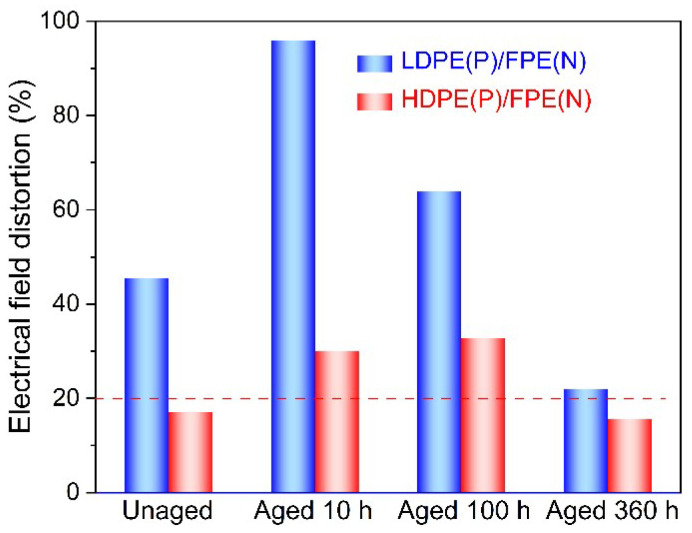
Electric field distortion of double-layer samples polarized for 40 min under −50 kV/mm.

From the electric field distortion, the maximum electric field of LDPE(P)/FPE(N) is −72.7 kV/mm, and the distortion rate is 45.5%. The maximum electric field of HDPE(P)/FPE(N) is −58.5 kV/mm, and the distortion rate is 17%. Furthermore, considering the influence of thermal aging, the electric field distortion is aggravated at the initial aging stage. Still, with the extension of thermal aging, the change of the internal microstructure tends to be stable. In contrast, the electric field distortion rate gradually decreases as shown in Figure 17.

## 4. Conclusions

This work investigates the thermal, mechanical, electrical, and space charge characteristics of thermally aged single-layer and double-layer PE insulation, contributing to establishing a life evaluation model of track cable insulation in future work. The conclusions are as follows:

(1) Both TGA and DSC are high-precision thermal analysis methods, and detailed thermal aging parameters were obtained in N_2_ and O_2_ atmospheres. Compared to additive-free LDPE, all three PE materials contain a certain amount of antioxidants. LDPE had the lowest melting point and thermal oxidation temperature region, while FPE had the highest melting point and crystallinity.
(2) The electric field distortion and space charge accumulation of HDPE film samples are the most obvious among the four unaged groups. However, the electric field distortion ratio of 360 h aged HDPE samples is much smaller than that of unaged HDPE, which may be related to the newly generated deep traps due to the thermal aging.
(3) A large amount of space charge accumulates at the unaged LDPE/FPE interface mainly due to the mismatching in DC conductivity between the two layers. After thermal aging, the space charge accumulation of the double-layer sample increases at first and decreases, which should be mainly attributed to the corresponding change of trap property and electrical conductivity.

## Figures and Tables

**Table 1:** Comparison of DSC OIT and TGA parameters of PE materials in the O_2_ atmosphere.

	DSC OIT in O_2_			TGA in O_2_		
	*T*_2_ (°C)	*T*_3_ (°C)	*T*_4_ (°C)	*T*_Init_ (°C)	*T*_Tang_ (°C)	*T*_Peak_ (°C)
Ref-LDPE	−	−	−	199.8	209.8	238.6
LDPE	249.7	255.9	267.6	255.7	260.3	267.8
FPE	245.5	252.8	260.5	253.7	258.5	265.3
HDPE	254.2	262.3	270.2	−	−	−

## Data Availability

The data presented in this study are available on request from the corresponding author.
